# Acute exercise mobilizes NKT-like cells with a cytotoxic transcriptomic profile but does not augment the potency of cytokine-induced killer (CIK) cells

**DOI:** 10.3389/fimmu.2022.938106

**Published:** 2022-09-14

**Authors:** Tiffany M. Zúñiga, Forrest L. Baker, Kyle A. Smith, Helena Batatinha, Branden Lau, Michael P. Gustafson, Emmanuel Katsanis, Richard J. Simpson

**Affiliations:** ^1^ School of Nutritional Sciences and Wellness, The University of Arizona, Tucson, AZ, United States; ^2^ University of Arizona Genetics Core, The University of Arizona, Tucson, AZ, United States; ^3^ Laboratory Medicine and Pathology, Mayo Clinic Arizona, Phoenix, AZ, United States; ^4^ Department of Pediatrics, The University of Arizona, Tucson, AZ, United States; ^5^ The University of Arizona Cancer Center, The University of Arizona, Tucson, AZ, United States; ^6^ Department of Immunobiology, The University of Arizona, Tucson, AZ, United States; ^7^ Department of Medicine, The University of Arizona, Tucson, AZ, United States; ^8^ Department of Pathology, The University of Arizona, Tucson, AZ, United States

**Keywords:** exercise immunology, cell therapy, physical activity, cancer, hematological malignancies, single cell RNA sequencing, cytotoxicity, donor lymphocyte infusions

## Abstract

CD3^+^/CD56^+^ Natural killer (NK) cell-like T-cells (NKT-like cells) represent <5% of blood lymphocytes, display a cytotoxic phenotype, and can kill various cancers. NKT-like cells can be expanded *ex vivo* into cytokine-induced killer (CIK) cells, however this therapeutic cell product has had mixed results against hematological malignancies in clinical trials. The aim of this study was to determine if NKT-like cells mobilized during acute cycling exercise could be used to generate more potent anti-tumor CIK cells from healthy donors. An acute exercise bout increased NKT-like cell numbers in blood 2-fold. Single cell RNA sequencing revealed that exercise mobilized NKT-like cells have an upregulation of genes and transcriptomic programs associated with enhanced anti-tumor activity, including cytotoxicity, cytokine responsiveness, and migration. Exercise, however, did not augment the *ex vivo* expansion of CIK cells or alter their surface phenotypes after 21-days of culture. CIK cells expanded at rest, during exercise (at 60% and 80% VO_2max_) or after (1h post) were equally capable of killing leukemia, lymphoma, and multiple myeloma target cells with and without cytokine (IL-2) and antibody (OKT3) priming *in vitro*. We conclude that acute exercise in healthy donors mobilizes NKT-like cells with an upregulation of transcriptomic programs involved in anti-tumor activity, but does not augment the *ex vivo* expansion of CIK cells.

## Introduction

Natural killer (NK) cell-like T-cells (NKT-like cells) are a heterogenous subset of effector T-cells (CD3^+^CD56^+^) that comprise ~1-5% of the peripheral blood lymphocyte compartment (1–3). These effector cells share phenotypic properties with NK-cells and can kill various cancer types in a non-MHC restricted manner (1, 2, 4), making them attractive candidates for allogeneic cell therapy. Blood NKT-like cells can be expanded *ex vivo* into a therapeutic cell product known as cytokine-induced killer (CIK) cells (2, 3), which have potent cytotoxic effects against multiple tumor cell lines *in vitro* (1, 5–7). The adoptive transfer of CIK cells *in vivo* reduces tumor burden by efficient homing mechanisms and long-term persistence (5, 7). Several clinical trials have demonstrated the feasibility and therapeutic efficacy of CIK cell adoptive therapy against both hematologic and solid malignancies, without treatment-induced severe adverse effects (8).

CIK cells are typically manufactured from patients or donors through lymphocytoapheresis or cord blood by the time-sensitive addition of an anti-CD3 monoclonal antibody and the cytokines IFN-γ and IL-2 *in vitro* (1, 3, 4). This expansion protocol generates a CIK cell population that is >90% CD3^+^ and 20-35% CD56^+^ within 14-28 days (6). However, CIK cells are sometimes difficult to expand due to the low numbers of CD3^+^CD56^+^ cells in peripheral blood (9). Moreover, despite moderate success in clinical trials to date, many patients with hematological malignancies do not enter remission after therapy or relapse (10, 11). Therefore, identifying a more feasible method to produce a superior CIK cell product could increase their utilization in the clinic.

A single bout of dynamic cardiovascular-based exercise (e.g. running, cycling, rowing) is an effective way to transiently increase the numbers of effector lymphocytes in peripheral blood 2-5-fold (12) evoking a preferential mobilization of effector lymphocytes that exhibit enhanced anti-tumor function (e.g. NK-cells, CD8^+^ T-cells, TCR-γδ T-cells) (12). In murine cancer models, these exercise-mobilized lymphocytes quickly travel to and infiltrate tumors and play a cytotoxic role in reducing tumor burden with exercise (13). We have previously shown that exercise-mobilized lymphocytes allow for the generation of superior therapeutic cell products including viral-specific T-cells, tumor antigen specific T-cells and TCR-γδ T-cells (14–16). Although exercise is known to mobilize large numbers of precursor CD3^+^CD56^+^ NKT-like cells into the circulation (17), it is not known if these mobilized cells display distinct anti-tumor transcriptomic signatures or if they can be used to improve the manufacture and potency of CIK cells.

The aim of this study was to characterize transcriptomic changes in exercise-mobilized NKT-like cells at the single cell level and determine if the mobilized cells can be used to enhance the *ex vivo* generation of CIK cells from healthy donors. We found that exercise-mobilized NKT-like cells have an upregulation of multiple transcriptomic programs associated with anti-tumor immune activity, including cytotoxicity, cytokine production and responsiveness, antigen binding and processing, and chemotactic capacity; however, the phenotype and potency of the expanded CIK cells against a range of hematological cancer cell lines *in vitro* was unaffected by exercise.

## Methods

### Participants

Ten healthy participants (five females, five males) aged 21-45 (31 ± 6.9) years were recruited for this study. Prior to their enrollment, each participant was screened and identified as ‘low-risk’ for cardiovascular disease in accordance with American Heart Association-American College of Sports Medicine criteria (18), were not taking medications with the exception of oral contraceptives, were physically active (score of >4 on the physical activity rating questionnaire) (19) and non-users of tobacco products. Participants were asked to abstain from alcohol, caffeine, and physical activity 24 h prior to each laboratory visit, as well as complete an 8-12 h overnight fast when only water was consumed. This was confirmed verbally upon their arrival to the laboratory. Additionally, participants were only permitted to consume water until all experimental procedures were completed during each visit. All participants provided written informed consent and the study was approved by the International Review Board at the University of Arizona. All laboratory procedures were performed between 6:00am-9:00am local time to minimize diurnal variation.

### Experimental design

Participants firstly completed a maximal graded exercise test on a cycling ergometer (Velotron, Quarq Technology, San Diego, CA) with real-time collection of respiratory gas exchange and heart rate (Quark CPET, COSMED, Pabona di Albona Laziale, Italy) to determine maximal oxygen uptake (VO_2max_). After a 5 min warm-up at 50 watts (W), resistance was increased by 15W every minute until the participant reached volitional exhaustion. Participants maintained a consistent cycling cadence throughout the entire exercise bout (≥60rpm) and rating of perceived exertion (RPE; Modified BORG scale (0–10)) was recorded during the final 15 s of each incremental stage. Linear regression plots were produced for each participant to determine cycling powers corresponding to 50%, 60%, 70%, and 80% VO_2max_ for the subsequent laboratory visit.

**Figure 1:**
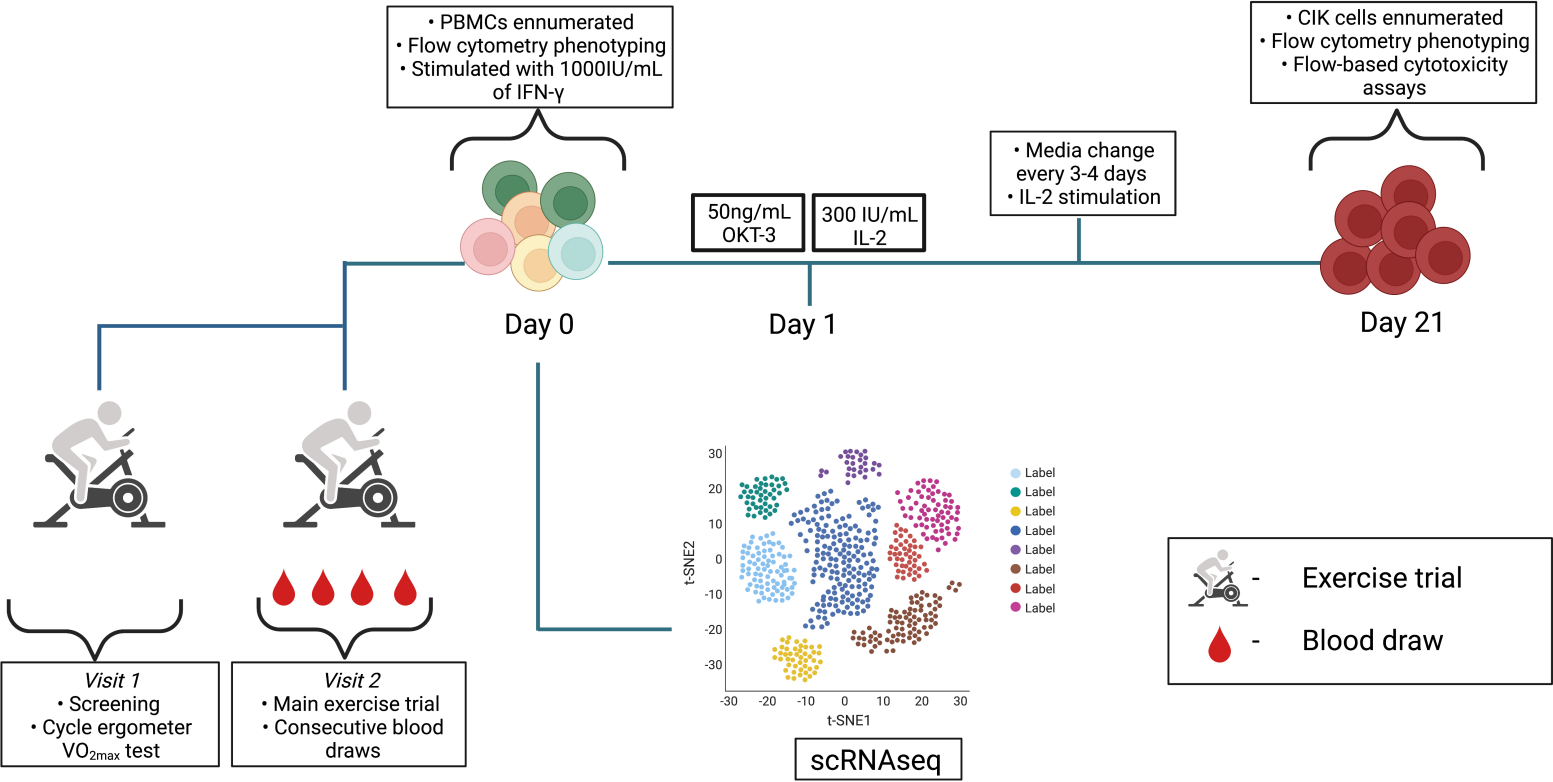
Schematic detailing the experimental design. Visit 1 and 2 consisted of exercise trials and serial blood draws were performed at rest, 60% VO_2max_, 80% VO_2max_, and +1H. PBMCs previously isolated from whole blood samples followed the CIK cell expansion protocol until day 21. scRNAseq was performed on resting PBMCs (*n* = 3). Diagram was created with BioRender.com.

Participants returned to the laboratory 1-3 weeks later to perform the main exercise trial. An indwelling catheter (BD, Franklin Lakes, NJ, USA) was placed inside an antecubital vein so that serial blood draws could be collected before, during, and after exercise. The catheter was flushed with a sterile isotonic saline solution after each blood draw and 3mL blood volume was drawn and discarded prior to collection of blood samples used for analysis. Blood samples were collected from each subject into a 6mL vacutainer collection tube containing acid-citrate dextrose (ACD) (BD Vacutainer^®^ blood collection tubes) for PBMC isolations or K_2_EDTA (BD Vacutainer^®^ blood collection tubes) for whole blood phenotyping. After collecting the resting blood sample, participants completed a 5 min warm-up at 50W and were then asked to cycle continuously for 20 min. The trial consisted of four incremental 5 min stages with power outputs corresponding to 50%, 60%, 70%, and 80% of the predicted VO_2max_. Participants again were asked to maintain a consistent cycling cadence throughout the entire exercise session (≥60rpm) and blood samples were collected from the IV catheter at the 60% and 80% cycling stages. Heart rate and oxygen uptake were measured continuously throughout the exercise trial and RPE was recorded during the final 15 s of every exercise stage. A final intravenous blood sample was collected 1 h (+1H) after exercise cessation. Overall, a schematic of the experimental design is shown in **Figure 1**.

### Immunophenotyping and lymphocyte subset enumeration

Whole blood samples were labeled with directly conjugated antibodies for multi-parameter flow cytometry to enumerate CD45^+^CD14^+^ monocytes and CD45^+^CD14^-^ lymphocytes subsets, as previously described (20). Briefly, 100µL of EDTA whole blood was incubated with the following antibodies CD8-VioBlue, CD14-VioGreen, CD3-FITC, CD4-PE, CD62L-PE, CD20-PerCP, CD45RA-PerCPVio770, CD45-APC, and CD56-APC-Vio770 (Miltenyi Biotec Inc., Gernany) for 30 min at room temperature and then lysed (RBC lysis buffer; eBioscience, San Diego, CA) for 20 min at room temperature followed by three wash cycles. For lymphocyte and monocyte enumeration, whole blood samples were labeled with CD14 and CD45 only, underwent a lyse-no-wash procedure and adjusted for the dilution factor. The total cell numbers of each lymphocyte subset were determined by multi-parameter flow cytometry (MACSQuant 10; Miltenyi Biotec Inc. Bergisch Gladbach, Germany) by multiplying the percentage of all lymphocytes expressing the surface markers of interest by the total lymphocyte count.

### Expansion of CIK cells

Peripheral blood mononuclear cells (PBMCs) were isolated from 6mL of ACD whole blood collected at rest, 60%, 80%, and +1H by density gradient centrifugation (Cytiva Ficoll-Paque™, Fisher Scientific, Hampton, NH) and cryopreserved until expansion. On Day 0, PBMCs were thawed and seeded at a concentration of 2-5 x 10^6^ cells/mL in a 6-well plate with RPMI-1640 (Sigma-Aldrich) consisting of 10% FBS (Sigma-Aldrich) and 1% penicillin streptomycin (Sigma-Aldrich). Additionally, the generation of CIK cells was primed with 1000IU/mL of IFN-γ and incubated at 37°C in a 5% CO_2_ humidified incubator for 24 h. The following day, 50ng/mL anti-CD3 (Miltenyi) and 300IU/mL IL-2 (Miltenyi) were added to the culture media to induce proliferation. Media was then changed every 3-4 days with the addition of 300IU/mL IL-2 and cells were seeded at a concentration of 1 x 10^6^ cells/mL. After 21 days, cells were harvested to determine number, phenotype, and function by flow cytometry. Expanded CIK cells were enumerated, and 2 x 10^5^ cells were labeled with appropriate combinations of the antibodies shown in **Supplementary Table 1**.

### CIK cell cytotoxicity assays

Flow cytometry-based cytotoxicity assays were performed to examine functionality of the expanded CIK cells. 3 x 10^6^ expanded CIK cells were stimulated for 1 h in media alone (RPMI-1640 with 10% FBS and 1% penicillin streptomycin) or media supplemented with 300IU/mL IL-2, 5ng/mL OKT-3, or a combination of IL-2+OKT-3 at 37°C in a 5% CO_2_ humidified incubator. CIK cells were then washed and recounted by flow cytometry. The leukemia cell line K562 (ATCC: CCL-243), the HLA-expressing (group 1 HLA-C ^*^0304,^*^0702) multiple myeloma cell line U266 (ATCC: TIB-196), and the Daudi (ATCC: CCL-213) Burkitt’s lymphoma cell line were used as target cells in the assays and maintained as previously described (20). 1 x 10^6^ target cells were labeled with anti-CD71-FITC for 30 minutes and subsequently counted by flow cytometry. 300IU/mL of IL-2 was added to washed CIK cells and co-cultured with CD71-labeled target cells at a 0:1 (spontaneous lysis control) or 5:1 (CIK cell: target cell) ratio in a 96-well plate in duplicate for 4 h. Flow cytometry was used to determine CIK cell cytotoxic activity by identifying propidium iodide (PI) positive cells among the CD71+ targets following methods we have previously described (21). Lytic activity was calculated as specific lysis (% total lysis - % spontaneous lysis).

### Single cell RNAsequencing (scRNAseq)

Isolated PBMCs from blood collected at rest, 80%, and +1H were resuspended in a PBS/RNAlater solution and delivered to the University of Arizona Genetics Core for single cell RNA sequencing (scRNAseq) analysis using the 10x Genomics platform. 5’ RNA whole transcriptome libraries were generated using the “10xGenomics Chromium Next GEM Single Cell 5’ reagents kit v2”, following recommended guidelines. The gene expression libraries were quantified, normalized, pooled, and sequenced on an Illumina NextSeq500 sequencer. FastQ files were converted into expression matrices using the “cellranger count” function provided by Cell Ranger (10x Genomics Cell Ranger 6.0.1) and unfiltered matrices were imported into R, version 4.1.0. Empty droplets were identified and removed using the emptyDrops function found in the DropletUtils package (22). Reads with a high percentage of mitochondrial content were identified and removed using the perCellQCMetrics function provided by scuttle (23). After these QC steps were performed, 9,323 genes remained for downstream analysis. In order to analyze and visualize gene expression on a per cell basis, Principle component analysis (PCA) and uniform manifold approximation and projection (UMAP) clustering was performed using Seurat, version 4.0.5, to identify NKT-like cells (24). Differentially expressed genes were then detected using the FindMarkers function, with a log2 fold cutoff of 0, in Seurat. For each differential expression analysis comparison, gene set enrichment analysis (GSEA), with a false discovery rate of (0.25), was performed and annotated to both Kyoto Encyclopedia of Genes and Genomes (KEGG) and Gene Ontology (GO) terms.

### Statistical analysis

All statistical analyses were completed using GraphPad Prism 8.0. All data are represented as the mean ± SD unless otherwise stated. Repeated measures ANOVA (RMANOVA) were used to analyze all cell number and flow cytometry surface expression levels (percentage or mean fluorescent intensity) across timepoints. RMANOVAs were used to analyze significant differences between resting and exercise-expanded CIK cells and their ability to kill target cell lines within stimulated conditions. Bonferroni test was applied to assess multiple comparisons for all RNAOVAs.

## Results

### Acute exercise mobilizes NKT-like cells with differentially expressed genes associated with cytotoxicity and tissue migration

**Figure 2:**
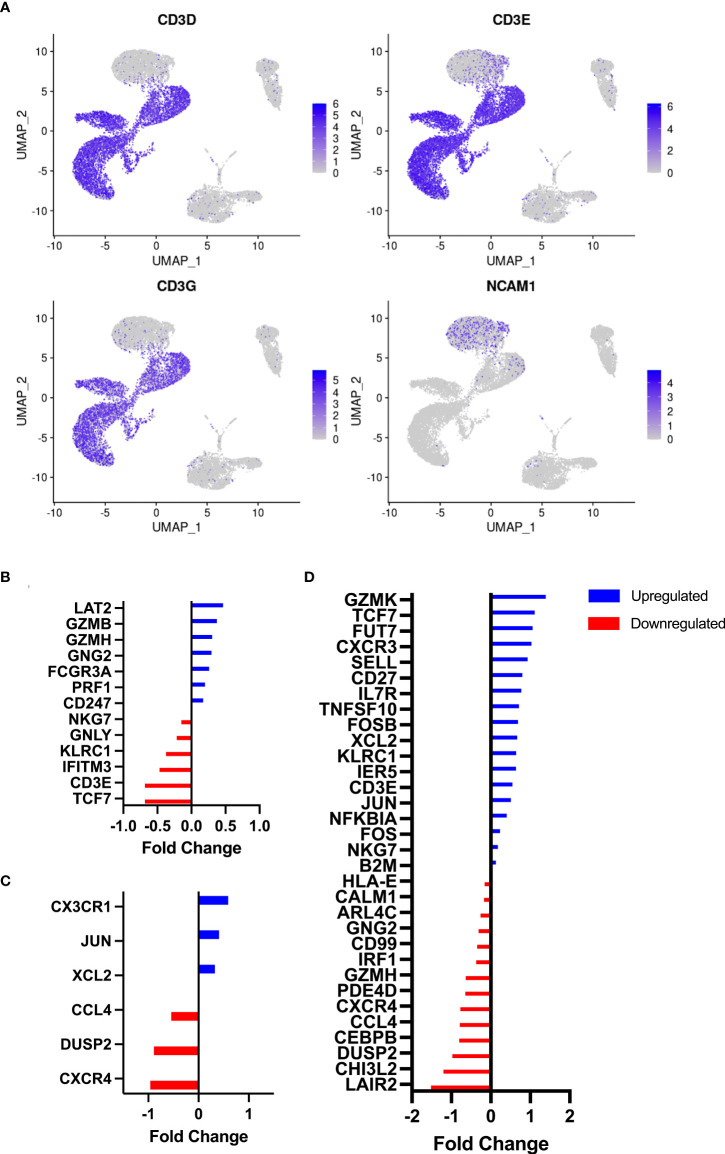
Identification of NKT-like cells by RNAseq and associated DEG analysis. **(A)** UMAP plots demonstrate clustering of CD3D, CD3E, and CD3G on resting PBMCs (*n* = 3). NKT-like cells were identified by the overlap of NK-cell marker NCAM1 and CD3 clusters. Differentially expressed genes on NKT-like cells were identified between timepoints, **(B)** Rest *vs* 80%, **(C)** Rest *vs* +1H, and **(D)** 80% *vs* +1H. Blue and red bars denote the upregulation and downregulation of DEGs, respectively. All are statistically significant (p<0.05).

As anticipated, numerous leukocyte subsets were mobilized to the peripheral blood compartment during exercise, including NKT-like cells which were elevated at both the 60% (*p* = 0.02*)* and 80% (*p* = 0.006) intensities compared to rest (**Table 1**). The numbers of NKT-like cells in blood at +1H fell below resting values (*p* = 0.01, **Table 1**). We then aimed to understand if acute exercise altered gene expression in NKT-like cells at the single cell level. NKT-like cells were identified on UMAP clusters by the NCAM1 cluster within the CD3 clusters of PBMCs (**Figure 2A**). Differentially expressed genes (DEGs) (25) within the NKT-like cell cluster were then compared among three timepoints (Rest, 80%, and +1H) (**Figure 2**, **Supplementary Table 2**). We found seven genes to be significantly upregulated and six to be significantly downregulated at the 80% intensity compared to rest (**Figure 2B**). Cytotoxic DEGs, such as GZMB, GZMH, PRF1 were upregulated at 80% compared to Rest (**Figure 2B**). Interestingly, KLRC1 which encodes for the inhibitory receptor NKG2A, was downregulated with exercise (26) (**Figure 2B**). Similarly, DEGs associated with cytotoxic function, including GZMK, TNFSF10, and NKG7 were upregulated when comparing 80% and +1H (**Figure 2D**, **Supplementary Table 1**). There were fewer enriched DEGs between Rest and +1H timepoints, however, most upregulated genes were associated with migratory potential (e.g. CXCR1, CCL4, CXCR4) (**Figure 2C**). Overall, upregulated genes in exercise-mobilized NKT-like cells were closely related to cytotoxicity and increased migratory potential.

**Table 1:** The total number (cells/μL) of lymphocytes, CD3+ T-cells, CD4+ T-cells, CD8+ T-cells, ‘double negative’ T-cells, NK-cells, NKT-like cells, B-cells, and monocytes present in peripheral blood before (rest), during (at 60% and 80% VO_2max_), and 1-hour post (+1H) exercise.

Leukocyte Subsets (cells/μL)	Rest	60%	80%	+1H
**Lymphocytes**	1745.02 ± 264.48	2381.62 ± 450.59***	3158 ± 606.80***	1463.44 ± 501.88
**CD3^+^ T-cells**	1231.52 ± 283.79	1564.41 ± 438.77*	1892.15 ± 584.70**	1108.79 ± 508.73
**CD4^+^ T-cells**	711.67 ± 244.42	802.21 ± 21*	920.48 ± 309.52**	633.10 ± 279.17
*Naïve*	280.47 ± 150.01	303.25 ± 170.79	341.97 ± 189.44*	210.48 ± 122.53
*CM*	167.17 ± 67.71	196.03 ± 74.33*	220.49 ± 139.47*	216.39 ± 118.67
*EM*	220.42 ± 107.65	256.51 ± 122.87	297.12 ± 139.47*	216.39 ± 118.67
*EMRA*	43.61 ± 45.79	46.42 ± 48.06	60.87 ± 63.44	54.93 ± 58.49
**CD8^+^T-cells**	407.67 ± 118.77	541.09 ± 211.68**	683.69 ± 290.51**	338.80 ± 136.17
*Naïve*	116.36 ± 54.56	134.35 ± 56.32	157.18 ± 65.03*	100.48 ± 48.53
*CM*	37.50 ± 18.15	48.88 ± 19.98***	56.32 ± 22.67***	33.62 ± 18.09
*EM*	184.03 ± 117.91	240.81 ± 193.72	323.34 ± 257.81*	142.36 ± 88.62*
*EMRA*	69.77 ± 66.55	207.07 ± 101.00*	146.87 ± 137.38*	62.36 ± 73.87
**CD4^-^CD8^-^ T-cells**	100.64 ± 60.00	140.22 ± 88.49**	173.47 ± 105.99**	78.12 ± 48.34*
**NK-cells**	317.57 ± 158.23	591.13 ± 336.13**	935.79 ± 495.16**	210.48 ± 113.89***
**NKT-like cells**	147.34 ± 67.31	208.48 ± 118.77*	254.21 ± 139.81**	106.04 ± 55.34*
**B-cells**	112.12 ± 30.06	147.58 ± 46.12	188.17 ± 74.49**	111.34 ± 52.11
**Monocytes**	332.731 ± 70.80	482.12 ± 117.73***	596.65 ± 135.09***	331.32 ± 108.75

uL – microliter, CM – central memory, EM – effector memory, NK – natural killer cells, NKT – natural killer cell-like T-cells.

CD4+ and CD8+ T-cell subsets are also provided. Differentiated T-cells are phenotyped as follows: naïve (CD45RA^+^CD62L^+^), central memory (CM; CD45RA^-^CD62L^+^), effector memory (EM; CD45RA^-^CD62^-^), terminally differentiated effector memory (EMRA; CD45RA^+^CD62L^-^). Significant differences compared to rest indicated by * (p < 0.05), ** (p < 0.01), *** (p < 0.001).

### NKT-like cells mobilized with exercise display gene sets enriched to cytotoxic, anti-viral, and cytokine signaling functions

**Figure 3:**
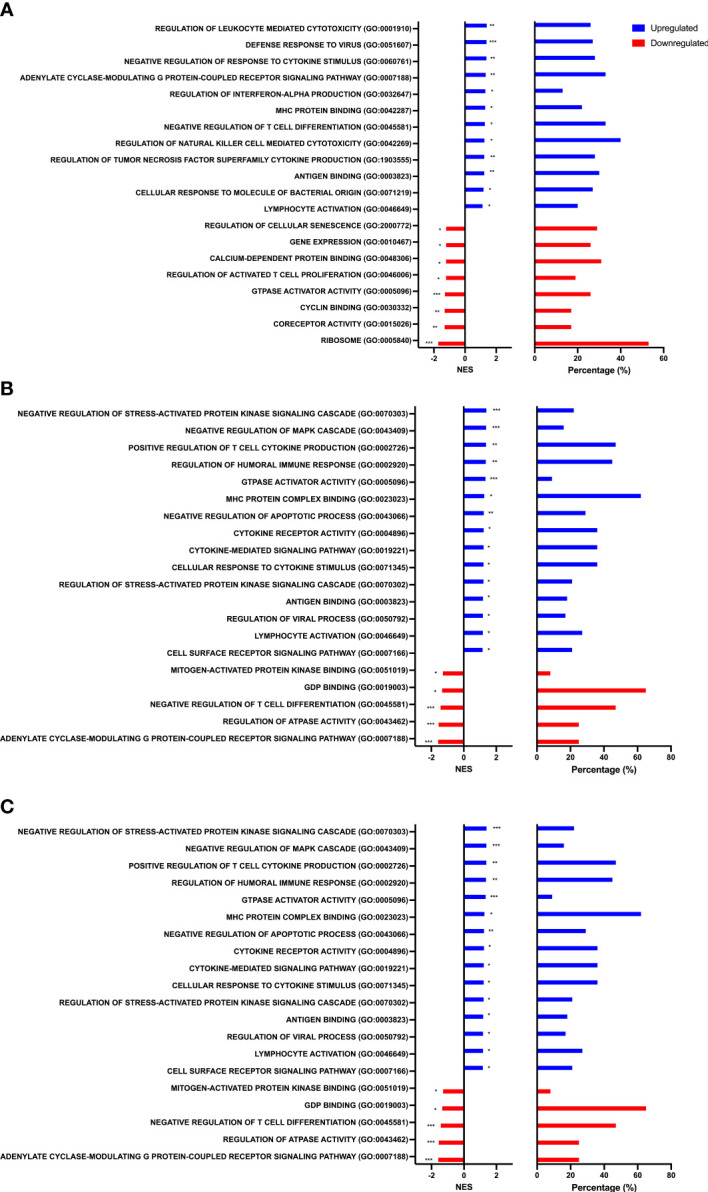
Gene set enrichment analysis performed using GO terms. Graphs show enriched upregulated (blue) and downregulated (red) pathways as well as the percentage of DEGs contributing to each mechanism between **(A)** Rest *vs* 80% **(B)** 80% *vs*. +1H, and **(C)** Rest *vs* +1H. Significance is indicated by * (*p* < 0.25), ** (*p* < 0.10), *** (*p* < 0.05).

**Figure 4:**
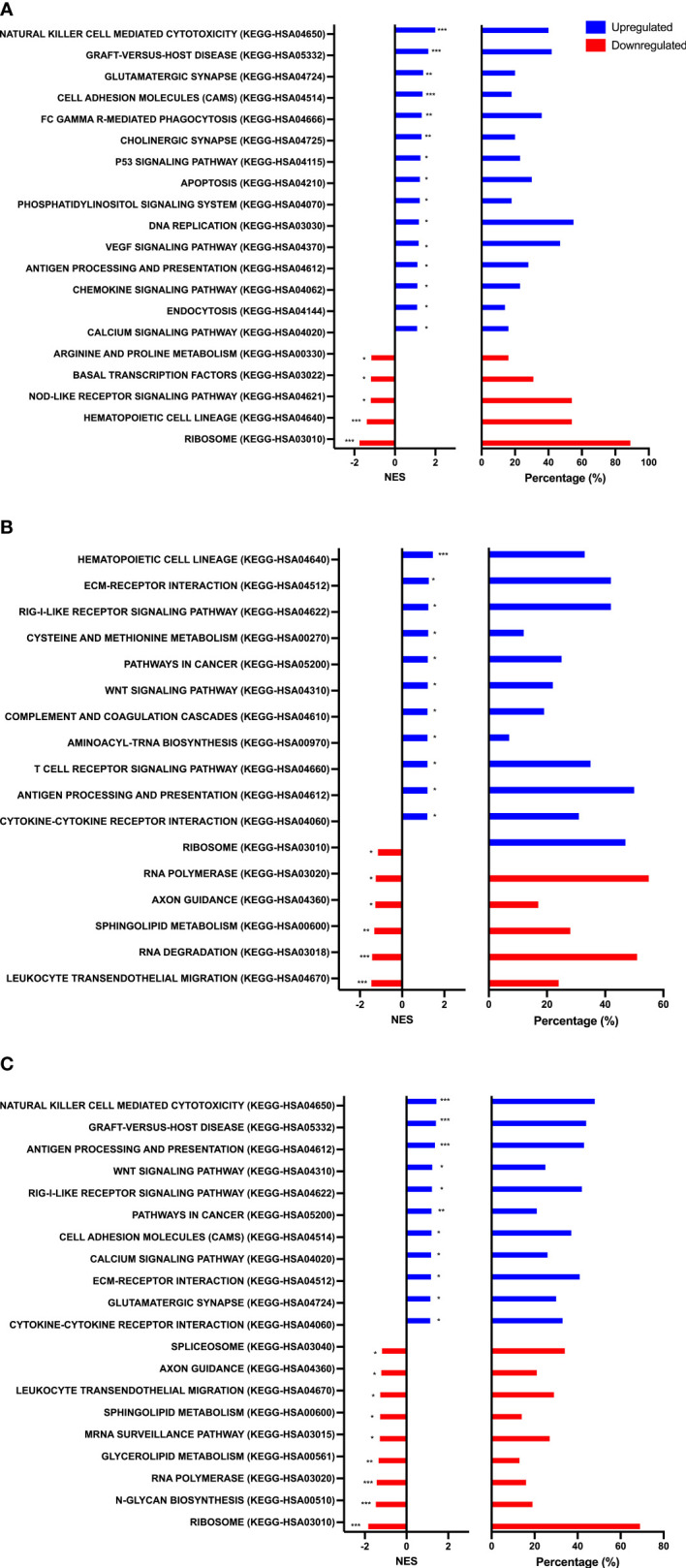
Gene set enrichment analysis performed using KEGG terms. Graphs show enriched upregulated (blue) and downregulated (red) pathways as well as the percentage of DEGs contributing to each mechanism between **(A)** Rest *vs* 80% **(B)** 80% *vs*. +1H, and **(C)** Rest *vs* +1H.Significance is indicated by * (*p* < 0.25), ** (*p* < 0.10), *** (*p* < 0.05).

**Figure 5:**
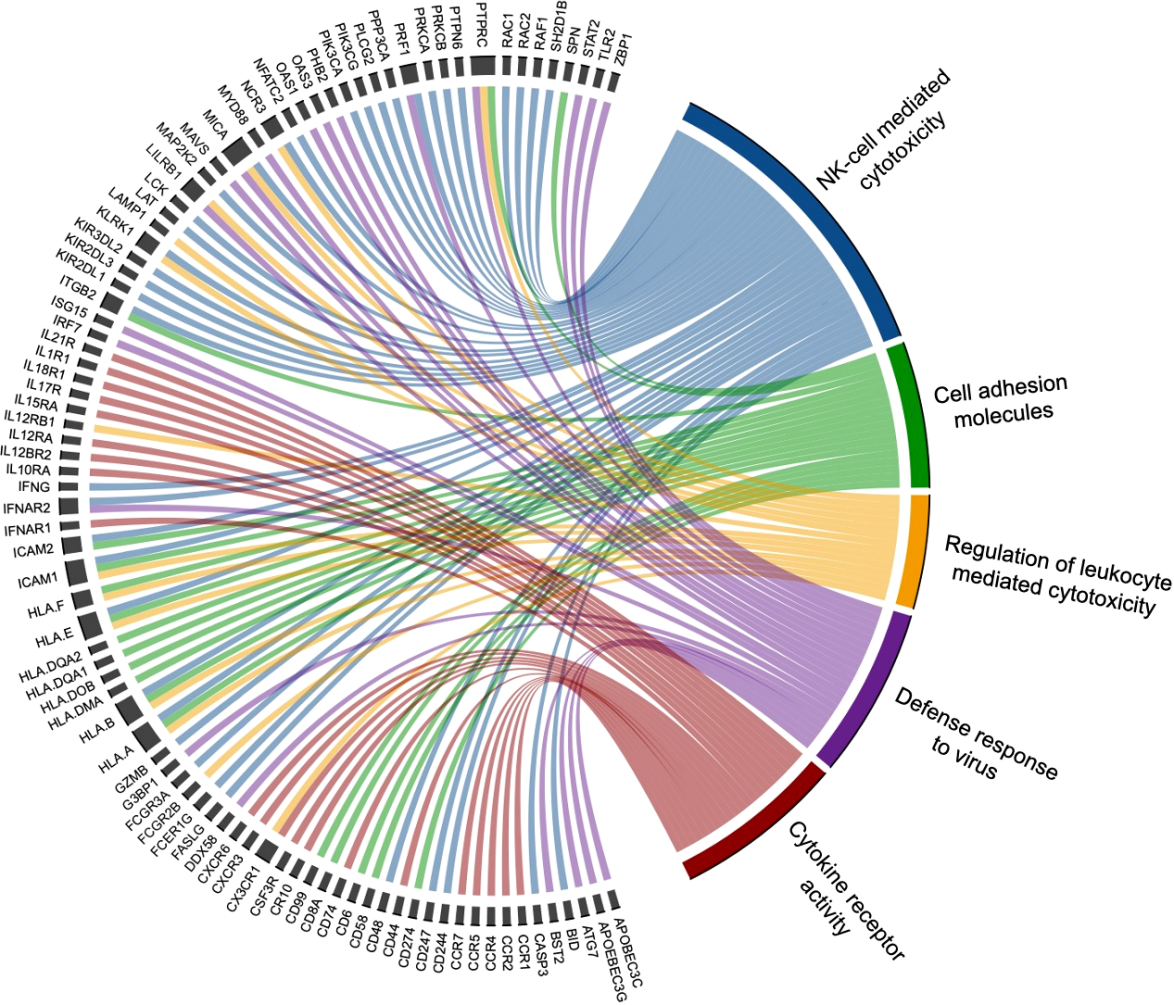
Leading edge genes are displayed in this chord diagram to indicate those that significantly drive the top selected GSEA pathways upregulated by exercise. The five biological processes with associated driving genes include NK-cell mediated cytotoxicity (blue), cell adhesion molecules (green), regulation of leukocyte mediated cytotoxicity (yellow), defense response to virus (purple), and cytokine receptor activity (red). Various genes are found to drive multiple pathways (e.g. perforin 1 [PRF1] drives NK-cell mediated cytotoxicity and defense to virus), while other genes are associated with one biological process.

To identify biological processes associated with our differential gene expression data, we performed functional annotation and gene set enrichment analysis (GSEA) using both GO and KEGG terms. We found 1,207 GO terms and 92 KEGG terms to be significantly (FDR < 0.25) enriched among our three sample time points (Rest, 80%, and +1H). We therefore selected the 20 most relevant terms in each comparison. Notably, from Rest to 80%, we found a significant upregulation of gene sets enriched to GO and KEGG terms associated with lytic function, including overall leukocyte mediated cytotoxicity, NK-cell mediated cytotoxicity, and FcγR-mediated phagocytosis (**Figures 3A** and **4A**). Gene sets associated with anti-viral responses were also upregulated, including defense response to viruses, regulation of interferon-alpha production, antigen presentation and processing, and antigen binding (**Figures 3A** and **4A**). Downregulated pathways were mainly enriched to intracellular processing mechanisms such as amino acid metabolism, calcium signaling, and ribosomal processes (**Figure 4A**). When comparing 80% to +1H, enriched gene sets indicated enhanced responses to stimuli, such as cytokine receptor activity, cytokine-mediated signaling, cytokine-cytokine receptor interaction, and cellular response to cytokine stimulus (**Figures 3B** and **4B**). Those that were downregulated included more intracellular processes such as ATPase activity, GDP binding, ribosome, and axon guidance (**Figures 3B** and **4B**). Similar processes such as enhanced cytotoxicity and overall lymphocyte signaling and activation were also observed when comparing Rest *vs*. +1H, with the additional upregulation of pathways in cancer (**Figures 3C** and **4C**). A summary of the leading-edge genes driving the gene set enrichment analyses for upregulation in NK-cell mediated cytotoxicity, cell adhesion molecules, regulation of leukocyte mediated cytotoxicity, defense response to virus, and cytokine receptor activity are also identified (**Figure 5**). Altogether, these analyses indicate that NKT-like cells mobilized during exercise exhibit transcriptomic profiles associated with greater cytotoxicity, anti-viral defense, and cytokine stimulus interaction.

### Acute exercise does not augment the *ex vivo* expansion of CIK cells

**Figure 6:**
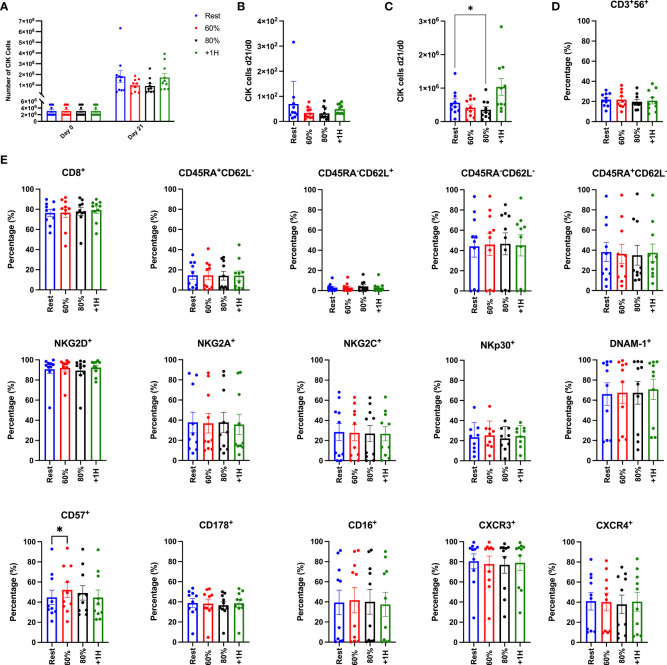
Enumeration and associated phenotypes of *ex vivo* expanded CIK cells. **(A)** The total number of PBMCs utilized for expansion on Day 0 and the total number of CIK cells generated in the expanded cell products after 21 days (*n* = 10). **(B)** The number of CIK cells generated at Day 21 divided by the number of CIK cells in the PBMC fraction at Day 0. **(C)** The number of CIK cells generated at Day 21 divided by the number of CIK cells in the NKT-cell fraction at Day 0. **(D)** The proportion of CD3^+^CD56^+^ expression on the total CIK cell population. **(E)** The percentage of surface markers expressed on the CD3^+^CD56 CIK cell population on day 21. Differentiated T-cells are phenotyped as follows: naïve (CD45RA^+^CD62L^+^), central memory (CM; CD45RA^-^CD62L^+^), effector memory (EM; CD45RA^-^CD62^-^), terminally differentiated effector memory (EMRA; CD45RA^+^CD62L^-^). Surface expression was determined by flow cytometry and error bars are represented as mean ± SEM. Significance is indicated by *(*p* < 0.05).

To determine if exercise mobilized lymphocytes would result in enhanced proliferation and cytotoxic activity of CIK cells, we expanded CIK cells from PBMCs collected at rest, during exercise (60% and 80%) and during exercise recovery (+1H) (**Figure 6**). Following the described 21-day expansion protocol, CIK cells increased 50-100-fold across all exercise timepoints (**Figure 6A**). Fewer total CIK cells, however, were generated on day 21 utilizing PBMCs collected at 60% and 80% compared to both Rest and +1H, although not significantly different (**Figure 6A**). Comparably, CIK cells generated after 21 days relative to the number of PBMCs on day 0 was lower from PBMCs collected at 60% and 80% compared to Rest and +1H (**Figure 6B**). Additionally, CIK cells generated after 21 days relative to the number of NKT-like cells in PBMC fractions on day 0 was significantly lowest at 80% compared to Rest (*p* = 0.01, **Figure 6C**). These results indicate that exercise-mobilized lymphocytes do not have enhanced proliferation *in vitro* and thus do not result in a higher yield of *ex vivo* generated CIK cells from healthy donors.

### Acute exercise does not affect the phenotype of *ex vivo* expanded CIK cells

Although exercise mobilized lymphocytes did not support enhanced *ex vivo* expansion of CIK cells, it was important to determine if the expanded products differed phenotypically. We performed a comprehensive phenotypic analysis on the expanded CIK cell products, focusing on surface markers related to cytotoxicity, homing, and activation (**Figure 6E**, **Supplementary Figure 1**). It has been shown previously that the percentage of CD56^+^ cells among expanded CIK cells is positively associated with their potency against leukemic targets (27–29). Although the proportion of CD56^+^ cells among our expanded CIK cell products (19-22%) is consistent with previous studies, exercise did not alter the composition of CD56+ cells among the expanded cell products. Furthermore, we found no differences in the expression of surface markers associated with CIK cell function, including NKG2D, DNAM-1, NKp30, and CD178 (FasL). We did, however, find that the terminal differentiation marker CD57^+^ was significantly elevated (*p* > 0.03) on CIK cells expanded from PBMCs collected during the 60% exercise intensity when compared to all other exercise timepoints (**Figure 6E**) (4, 30). Overall, these findings indicate that exercise does not modify the surface phenotypes of expanded CIK cell products.

### Acute exercise does not augment *in vitro* cytotoxicity of expanded CIK cells

**Figure 7:**
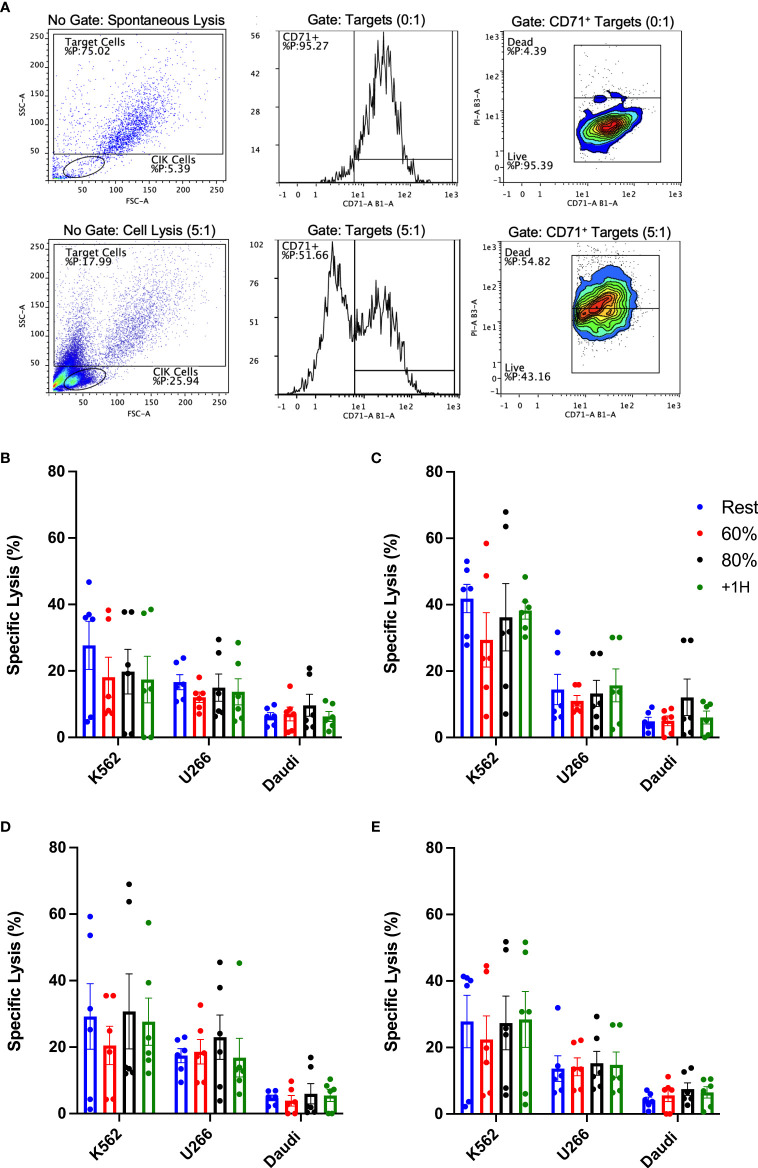
Exercise and cytokine stimulation does not augment anti-tumor activity of CIK cells against various cell targets *in vitro.* Cytotoxicity of CIK cells was assessed *via* flow cytometry-based assays against K562, U266, and Daudi cells at a E:T ratio of 5:1 (*n* = 6). **(A)** Representative flow cytometry plots illustrate the gating strategy utilized to determine specific lysis (CD71^+^/PI^+^). Expanded CIK cells were stimulated in **(B)** media alone or media supplemented with **(C)** 300IU/mL IL-2, **(D)** 5 ng/mL OKT-3, or **(E)** a combination of IL-2 and OKT-3. Error bars are represented as mean ± SEM. There were no significant differences found in lytic function between exercise timepoints.

Despite no discernible phenotypic differences between CIK cells expanded using resting and exercise mobilized lymphocytes, it was important to test for potential differences in cytotoxic function. The cytotoxic activity of expanded CIK cells was determined *in vitro* against three tumor cell lines, K562, U266, and Daudi (**Figure 7**). We also examined the effect of adding IL-2, OKT-3, or both (IL-2 + OKT-3) *in vitro* (**Figures 7B–D**), as they can augment the cytotoxic function of CIK cells (28, 31). Overall, CIK cells seemed to exhibit higher killing against K562 compared to U266 and Daudi cells (**Figure 7A**). However, CIK cell lytic activity against all three tumor cell lines was similar across the exercise time points (**Figure 7A**). Adding IL-2 or OKT-3 to the cultures did not increase the potency of the CIK cytolytic activity in cells expanded during or after exercise (p>0.05). These findings indicate that, in addition to not altering the phenotype of the expanded products, CIK cells expanded using exercise mobilized lymphocytes do not differ in their ability to kill hematologic target cells *in vitro*.

## Discussion

CIK cells are candidates for adoptive immunotherapy due to their accessibility from donors, manufacturing simplicity, high proliferative capacity, and non-MHC-restricted anti-tumor function. While CIK cells have been met with mixed results in clinical trials (10, 11), several groups have attempted to optimize their expansion and potency (32, 33). We attempted to use acute exercise as a simple intervention to augment the manufacture of CIK cells from healthy donors. We report, for the first time, that NKT-like cells, considered precursors to CIK cells, mobilized to the blood with exercise display transcriptomic profiles associated with enhanced cytotoxicity, anti-viral defense, and cytokine responsiveness. Despite this, exercise mobilized lymphocytes did not alter the phenotype or cytotoxic function of CIK cells expanded in culture over a 21-day period.

Exercise-mobilized leukocytes have been purported to offer a better source of therapeutic cell products for the treatment of both cancers and viral infections (34). Several studies, mostly from our group, have found that exercise-mobilized cells allow for improvements in the *ex vivo* manufacture of viral-specific T-cells, tumor-antigen specific T-cells and TCR-γδ T-cell products (14–16). As exercise preferentially mobilizes effector lymphocytes, including T-cells with a CD3^+^CD56^+^ phenotype (35), we hypothesized that exercise would also have adjuvant effects in manufacturing CIK cells that could be utilized more effectively in treating blood cancers. We found that NKT-like cells were mobilized to blood during both moderate (60% VO_2max_) and vigorous (80% VO_2max_) intensity exercise, falling below resting values 1 h later. As lymphocytes mobilized with exercise have phenotypes associated with cytotoxicity, migration, and antigen experience (16, 20), we performed a deep transcriptomic analysis of NKT-like cells mobilized with exercise at the single cell level. Various genes associated with cytotoxicity such as GZMB, GZMH, and PRF1 were upregulated in NKT-like cells mobilized with exercise. Similarly, expression of genes involved in effector lymphocyte inhibition such as KLRC1 were downregulated. These findings indicate that NKT-like cells mobilized with exercise have the potential for enhanced cytotoxic function compared to those in resting blood. Indeed, gene set enrichment analysis to GO and KEGG terms revealed an upregulation in processes associated with lytic function, anti-viral responses, and cytokine receptor activation. These findings indicate that exercise could be used to enhance both the function and redistribution of NKT-like cells, which could have implications for cancer patients that exhibit distinct functional impairments within this immune cell population (36–38). Indeed, dysfunction in NKT-like cells have been correlated with poor prognosis and patient outcomes in gastric cancer (39) as well as CLL progression (39, 40). The use of therapeutics such as tyrosine kinase inhibitors and immune checkpoint inhibitors have been proposed to restore functionality of NKT-like cells and increase cytotoxicity (37, 38). Whether exercise, either alone or in combination with other therapeutics, can increase the function of NKT-like cells in cancer patients that elicit improvements in prognosis or outcomes remains to be determined.

The adoptive transfer of donor-derived CIK cells alone or in combination with other immunotherapies have been found to significantly improve survival and quality of life in patients with solid tumors (8). Conversely, their effectiveness against hematological malignancies such as AML and CML have been less successful (10, 41). NKT-like cells mobilized to blood during exercise displayed transcriptomic profiles indicative of increased anti-tumor function. Despite this, we did not find improvements in phenotype or potency when exercise mobilized lymphocytes were used as the source of our expanded CIK cell products. Overall, CIK cells expressed CD8 and were characterized as effector memory or terminally differentiated effector memory cells, corroborating previous findings (27). Interestingly, CIK cells expanded from the 60% exercise intensity stage had greater CD57^+^ surface expression compared to all other time points. CD57 expression reflects NK-cell maturation and typically increases with age or chronic infections, such as CMV (42). While cells that express CD57 still sustain their cytotoxic abilities, proliferative capacity and cytokines responsiveness is reduced (42). This may explain the slight decrease in the number of CIK cells we were able to expand when using exercise-mobilized lymphocytes. Moreover, our functional annotation and enrichment analysis revealed a downregulation of gene sets associated with activated T-cell proliferation on NKT-like cells mobilized with exercise, indicating that CIK precursor cells mobilized with exercise, despite having increased cytotoxic potential, may have reduced proliferative capacity. Although NKT-like cells mobilized to blood with exercise exhibited an upregulation of individual genes and enriched gene sets associated with cytotoxicity, the *in vitro* cytotoxicity of expanded CIK cells against three hematological target cell lines was unaffected by exercise. Why acute exercise can boost the *ex vivo* manufacture of certain lymphocyte products (e.g. TCR-γδ T-cells, VSTs) but not CIK cells is yet to be determined. It is possible that consistent stimulation with high concentrations of cytokines over an extended period may override any beneficial effect of using exercise-mobilized lymphocytes with altered phenotypic and transcriptomic profiles. Despite this, the *in vivo* effects of exercise on NKT-like cells and other lymphocytes could have adjuvant effects for ‘untouched’ adoptive cell therapies, including standard donor lymphocyte infusions whereby lymphocytes are collected from donors and transferred to patients without any *ex vivo* manipulation (43), but this has yet to be determined.

A limitation of the present study includes the use of only one protocol to expand CIK cells. Although expansions were successful across all exercise timepoints, several studies have modified culture conditions to improve the proliferation and functional capacity of CIK cell products (32, 33, 44–46). For example, previous groups have determined that activated CIK cells stimulated with IL-15 exhibit enhanced cytotoxic potential against ALL and lymphoma cell lines (33, 46), therefore there is a possibility that exercise could have boosted CIK cell manufacture using different stimuli and cell culture conditions. Additionally, other groups have hypothesized that while CD3^+^CD56^+^ cells maintain expression and functionality after expansion, outgrowth of CIK cells may stem from CD3^+^CD56^-^CD4^-^CD8^+^ and CD3^+^CD56^-^CD4^-^CD8^-^ precursors (3, 27). Consequently, the exercise-induced transcriptomic shifts observed in NKT-like cells reported here may not have translated to the performance of the expanded CIK cell products. Moreover, it is possible that our small sample size prevented us from observing an exercise effect on the numbers and potency of expanded CIK cells. However, given that a statistical power analysis estimated a sample size of 280 individuals, any positive effect of exercise is likely to be small. It is also possible that exercise expanded CIK cells will be more potent against other hematologic cancer cell lines not used in this study.

In summary, this is the first study to provide a detailed single cell transcriptomic analysis of NKT-like cells mobilized to blood with exercise, revealing that exercise mobilized cells display transcriptomic profiles associated with enhanced lytic function, migration, cytokine signaling interaction, and overall activation. These findings add to the large body of evidence indicating that exercise mobilizes effector lymphocytes with potent anti-tumor potential (34). While exercise did not alter the phenotype or increase the potency of *ex vivo* generated CIK cells, future studies may consider the effects of exercise on NKT-like cells that may increase the effectiveness of unmanipulated donor lymphocyte infusion products for patients with hematological malignancies.

## Data availability statement

The data presented in the study are deposited in the GEO repository, accession number GSE212740.

## Ethics statement

The studies involving human participants were reviewed and approved by University of Arizona IRB. The patients/participants provided their written informed consent to participate in this study.

## Author contributions

TMZ, RJS and EK developed the theoretical framework, working hypotheses, and designed the study. TMZ, FLB, KAS and HB performed laboratory experiments. TMZ, FLB, and BL analyzed results. TMZ, RJS, FLB, and EK interpreted data. TMZ and RJS wrote the manuscript with contributions from FLB, KAS, HB, BL, MPG, and EK. The statistical analyses were performed by TMZ, BL and FLB. The overall study was supervised by RJS. All authors contributed to the article and approved the submitted version.

## Conflict of interest

The authors declare that the research was conducted in the absence of any commercial or financial relationships that could be construed as a potential conflict of interest.

## Publisher’s note

All claims expressed in this article are solely those of the authors and do not necessarily represent those of their affiliated organizations, or those of the publisher, the editors and the reviewers. Any product that may be evaluated in this article, or claim that may be made by its manufacturer, is not guaranteed or endorsed by the publisher.

## Supplementary material

Supplementary Table 1 List of directly conjugated antibodies used to label expanded CIK cells on day 21 *via* flow cytometry.

Supplementary Table 2 Differentially expressed genes in NKT-like cells in response to exercise as determined by single cell RNAseq. GeneCards^®^: the human gene database was utilized to identify gene name and function (26). Upwards arrows (↑) indicate differentially expressed gene was upregulated between timepoints; downwards arrow (↓) indicates differentially expressed gene was downregulated between timepoints; double-sided arrows (↔) indicate there was no significant change in gene expression between timepoints.

Supplementary Figure 1 All graphs represent percentage of surface marker expression on the CD3^+^CD56^+^ CIK cell population on day 21 (*n* = 10). Expression was determined by flow cytometry and error bars are represented as mean ± SEM. Significance is indicated by * (*p* < 0.05).
