# Supplementary material for: Acute exercise mobilizes NKT-like cells with a cytotoxic transcriptomic profile but does not augment the potency of cytokine-induced killer (CIK) cells
Source: Front Immunol. 2022 Sep 14;13:938106. doi: 10.3389/fimmu.2022.938106 (PMC9519182; doi:10.3389/fimmu.2022.938106)
Supplement: Supplementary Table 1 — List of directly conjugated antibodies used to label expanded CIK cells on day 21 via flow cytometry. [file Table_1.docx]

Supplementary Table 1. List of directly conjugated antibodies used to label expanded CIK cells on day 21 via flow cytometry.

| **Name** | **Alternate Name** | **Fluoroflour** | **Company** |
| --- | --- | --- | --- |
| CD8 | -- | VioBlue | Miltenyi |
| CD16 | -- | VioBlue | Miltenyi |
| CC195 | CCR5 | BV421 | BioLegend |
| CD253 | TRAIL | BV421 | BD BioSiences |
| CD14 | -- | VioGreen | Miltenyi |
| CD3 | -- | VioGreen | Miltenyi |
| CD4 | -- | FIT-C | Miltenyi |
| CD159a | NKG2A | VioBright FIT-C | Miltenyi |
| CD184 | CXCR4 | VioBright FIT-C | Miltenyi |
| CD62L | L-selectin | PE | Miltenyi |
| CD314 | NKG2D | PE | Miltenyi |
| CD183 | CXCR3 | PE | Miltenyi |
| HLA-ABC | -- | PE | Miltenyi |
| CD45 | -- | PerCP-Vio 700 | Miltenyi |
| CD279 | PD-1 | PerCP-Vio 700 | Invitrogen |
| CD337 | NKp30 | PerCP-Vio 700 | Miltenyi |
| CD45RA | -- | PE-Vio 770 | Miltenyi |
| CD178 | FasL | PE-Vio 770 | Miltenyi |
| CD197 | CCR7 | PE-Vio 770 | Miltenyi |
| CD159c | NKG2C | PE-Vio 770 | Miltenyi |
| CD56 | -- | APC | Miltenyi |
| CD226 | DNAM-1 | APC | Miltenyi |
| CD57 | -- | APC | Miltenyi |
| HLA-DR/DP/DQ | -- | APC | Miltenyi |
| TCR-γδ | -- | APC-Vio 770 | Miltenyi |
