# Supplementary material for: Acute exercise mobilizes NKT-like cells with a cytotoxic transcriptomic profile but does not augment the potency of cytokine-induced killer (CIK) cells
Source: Front Immunol. 2022 Sep 14;13:938106. doi: 10.3389/fimmu.2022.938106 (PMC9519182; doi:10.3389/fimmu.2022.938106)
Supplement: Supplementary Table 2 — Differentially expressed genes in NKT-like cells in response to exercise as determined by single cell RNAseq. GeneCards®: the human gene database was utilized to identify gene name and function (26). Upwards arrows (↑) indicate differentially expressed gene was upregulated between timepoints; downwards arrow (↓) indicates differentially expressed gene was downregulated between timepoints; double-sided arrows (↔) indicate there was no significant change in gene expression between timepoints. [file Table_2.docx]

Supplementary Table 2. Differentially expressed genes in NKT-like cells in response to exercise as determined by single cell RNAseq. GeneCards^®^: the human gene database was utilized to identify gene name and function [26]. Upwards arrows (↑) indicate differentially expressed gene was upregulated between timepoints; downwards arrow (↓) indicates differentially expressed gene was downregulated between timepoints; double-sided arrows (↔) indicate there was no significant change in gene expression between timepoints.

| **Gene** | **Name** | **Function**  *GeneCards^â^* | **Rest vs 80%** | **80% vs +1H** | **Rest vs**  **+1H** |
| --- | --- | --- | --- | --- | --- |
| LAT2 | Linker for activation of T-cells family member 2 | Adaptor/scaffolding molecule to recruit critical signaling molecules into the receptor signaling complex | ↑ | ↔ | ↔ |
| GZMB | Granzyme B | Secreted by natural killer cells and cytotoxic T-lymphocytes to induce target cell apoptosis | ↑ | ↔ | ↔ |
| GZMH | Granzyme H | Constitutively expressed in natural killer cells, induces target cell death by directly cleaving substrates in pathogen-infected cells | ↑ | ↔ | ↓ |
| GNG2 | G Protein subunit gamma 2 | Encodes for one of the gamma subunits of a guanine nucleotide-binding protein that are involved in signaling mechanisms across membranes | ↑ | ↔ | ↓ |
| FCGR3A | Fc region receptor III-A | Antibody dependent cellular mediated cytotoxicity and antibody dependent enhancement of virus infections | ↑ | ↔ | ↔ |
| PRF1 | Perforin-1 | Encodes for glycoprotein responsible for pore formation in cell membranes of target cells | ↑ | ↔ | ↔ |
| CD247 | T-cell surface glycoprotein CD3 zeta chain | Couples antigen recognition to several intracellular signal-transduction pathways | ↑ | ↔ | ↔ |
| NKG7 | Natural killer cell granule 7 | Expression in NK cells is critical for controlling cancer initiation growth and metastasis | ↓ | ↔ | ↑ |
| GNLY | Granulysin | Member of the saposin-like-protein family and is located in cytotoxic granules of T-cells that are released upon antigen stimulation | ↓ | ↔ | ↔ |
| KLRC1 | Killer cell lectin like receptor C1 | Gene encodes protein belongs to the NKG2 family, preferentially expressed on NK cells; immune inhibitory receptor involved in non-self recognition; can recognize non-classical MHC molecules in complex with KLRD1 | ↓ | ↔ | ↔ |
| IFITM3 | Interferon-induced transmembrane | Encodes for proteins that restrict cellular entry by diverse viral pathogens, IFN-induced antiviral protein that disrupts cholesterol balance | ↓ | ↔ | ↔ |
| CD3E | CD3-epsilon | Encodes for CD3-epsilon polypeptide, forms with other CD heterodimers to form T-cell receptor CD3 complex | ↓ | ↔ | ↔ |
| TCF7 | T-cell factor/lymphoid enhancer-binding factor 7 | Transcriptional activator involved in lymphocyte differentiation, necessary for survival of CD4+ and CD8+ immature thymocytes | ↓ | ↔ | ↑ |
| CXCR1 | C-X-C motif chemokine receptor 1 | Receptor to IL-8, a powerful neutrophil chemotactic factor | ↔ | ↑ | ↔ |
| JUN | Jun-Proto-Oncogene, AP-1 transcription factor subunit | Putative transforming gene of avian sarcoma virus 17; encodes protein that interacts directly with target DNA sequences to regulate gene expression | ↔ | ↑ | ↔ |
| XCL2 | X-C motif chemokine ligand 2 | Chemotactic activity for lymphocytes; related pathways include peptide-ligand binding receptors and signaling by GPCR | ↔ | ↑ | ↔ |
| CCL4 | C-C motif chemokine ligand 4 | Mitogen-inducible monokine, one of major HIV-suppressive factors produced by CD8+ T-cells | ↔ | ↓ | ↓ |
| DUSP2 | Dual specificity phosphatase 1 | Encoded protein desphosphorylates MAP kinase MAPK1/ERK2; involved in negative regulation of cellular proliferation | ↔ | ↓ | ↓ |
| CXCR4 | C-X-C motif chemokine receptor 4 | Encodes for CXC receptor specific for SDF-1 binding; increases intracellular calcium ion levels and enhances MAPK1/3 activation; regulation of cell migration | ↔ | ↓ | ↓ |
| GZMK | Granzyme K | Encodes from cytoplasmic granules of cytotoxic lymphocytes (e.g. CTLs and NK-cells); lacks consensus sequences for N-glycosylation present in other granzyme proteins | ↔ | ↔ | ↑ |
| FUT7 | Fucosyltransferase 7 | Encodes Golgi stack membrane protein that is involved in creation of sialyl-Lewis X antigens | ↔ | ↔ | ↑ |
| CXCR3 | C-X-C motif chemokine receptor 3 | Encodes GPCR that binds to CXCL9, CXCL10, and CXCL11; primarily expressed on activated T-cells and NK cells; regulates leukocyte trafficking; promotes Th1 cell maturation | ↔ | ↔ | ↑ |
| SELL | Selectin L | Encodes a cell surface adhesion molecule that belongs to a family of adhesion/homing receptors | ↔ | ↔ | ↑ |
| CD27 | CD27 | Member of the TNF-receptor superfamily, required for long-term maintenance of T-cell immunity | ↔ | ↔ | ↑ |
| IL7R | Interleukin 7 receptor | Receptor requires IL2RG, plays a critical role in VDJ recombination during lymphocyte development | ↔ | ↔ | ↑ |
| TNFS10 | Tumor necrosis factor superfamily member 10 | Encoded protein preferentially induces apoptosis in transformed and tumor cells | ↔ | ↔ | ↑ |
| FOSB | FosB proto-oncogene, AP-1 transcription factor | Fos gene consists of four members, encodes for zipper proteins that can dimerize with Jun family proteins; Fos proteins associated with cell proliferation, differentiation, and transformation | ↔ | ↔ | ↑ |
| IER5 | Immediate early response 5 | Plays role as a transcription factor by mediating positive regulation of chaperone genes during heat shock response; involved in regulation of cell proliferation and resistance to thermal stress | ↔ | ↔ | ↑ |
| NFKBIA | NF-kappa-B inhibitor alpha | Encoded proteins interacts with REL dimers to inhibit NFkB complexes which are involved inflammatory responses | ↔ | ↔ | ↑ |
| FOS | Fos proto-oncogene, AP-1 transcription factor | Fos gene consists of four members, encodes for zipper proteins that can dimerize with Jun family proteins; Fos proteins associated with cell proliferation, differentiation, and transformation | ↔ | ↔ | ↑ |
| B2M | Beta-2-microglobulin | Encodes component of MHC Class I molecules present on nucleated cells | ↔ | ↔ | ↑ |
| HLA-E | Major histocompatibility complex, class-I, E | Non-classical MHC Ib molecule involved in immune self-non-self recognition | ↓ | ↔ | ↔ |
| CALM1 | Calmodulin 1 | Mediates the control of cardiac ion channels through calcium-induced activation | ↓ | ↔ | ↔ |
| ARL4C | ADP ribosylation factor like GTPase 4C | Member of ADP-ribosylation factor family of GTP binding proteins; may be involved in cholesterol transport | ↓ | ↔ | ↔ |
| CD99 | CD99 | Encodes cell surface glycoprotein involved in leukocyte migration, T-cell adhesion, and T-cell death by a caspase-independent pathway | ↔ | ↔ | ↓ |
| IRF1 | Interferon regulatory factor 1 | Transcriptional regulator and tumor suppressor; activator of genes involved in innate and acquired immune responses; plays role in body’s response to virus/bacteria, cell proliferation, apoptosis, DNA damage response | ↔ | ↔ | ↓ |
| PDE4D | Phosphodiesterase 4A | Belongs to PDE family, hydrolyzes the second messenger cAMP, a mediator of cellular responses to extracellular signals | ↔ | ↔ | ↓ |
| CEBPB | CCAAT Enhancer Binding Protein Beta | Transcription factor important in the regulation of genes involved in the immune and inflammatory responses | ↔ | ↔ | ↓ |
| CHI3L2 | Chitinase 3 like 2 | Encoded protein lacks chitinase activity; is secreted and involved in cartilage biogenesis | ↔ | ↔ | ↓ |
| LAIR2 | Leukocyte associated immunoglobulin like receptor 2 | Modulates innate immune response; associated with pathways of class I MHC mediated antigen processing and presentation | ↔ | ↔ | ↓ |
