# Supplementary figures and images for: Acute exercise mobilizes NKT-like cells with a cytotoxic transcriptomic profile but does not augment the potency of cytokine-induced killer (CIK) cells
Source: Front Immunol. 2022 Sep 14;13:938106. doi: 10.3389/fimmu.2022.938106 (PMC9519182; doi:10.3389/fimmu.2022.938106)

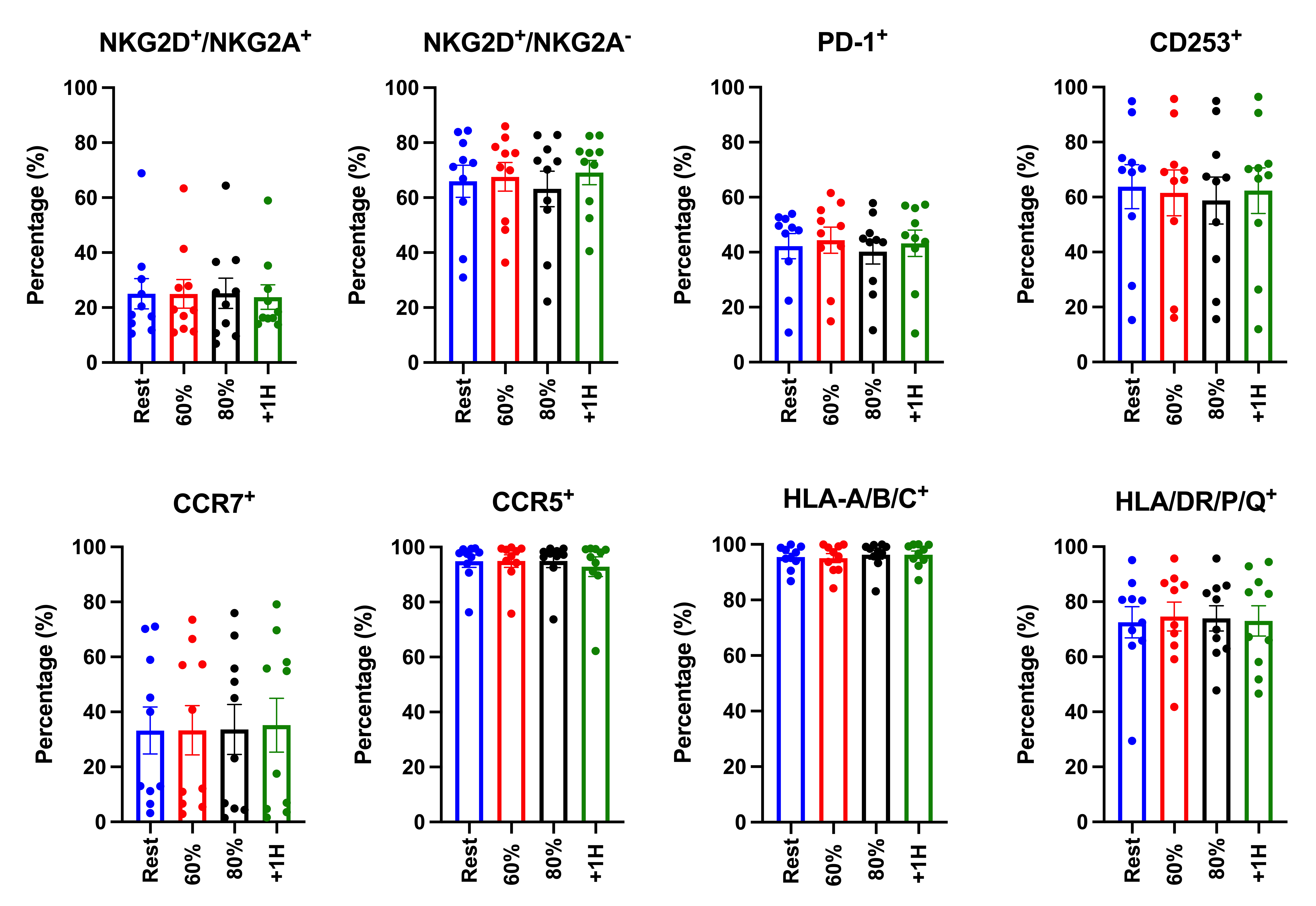

Supplement: Supplementary Figure 1 — All graphs represent percentage of surface marker expression on the CD3+CD56+ CIK cell population on day 21 (n = 10). Expression was determined by flow cytometry and error bars are represented as mean ± SEM. Significance is indicated by * (p < 0.05). [file Image_1.tiff]
